# Evolution and emergence of *Mycobacterium tuberculosis*

**DOI:** 10.1093/femsre/fuae006

**Published:** 2024-02-14

**Authors:** Mickael Orgeur, Camille Sous, Jan Madacki, Roland Brosch

**Affiliations:** Institut Pasteur, Université Paris Cité, CNRS UMR 6047, Unit for Integrated Mycobacterial Pathogenomics, 75015 Paris, France; Institut Pasteur, Université Paris Cité, CNRS UMR 6047, Unit for Integrated Mycobacterial Pathogenomics, 75015 Paris, France; Institut Pasteur, Université Paris Cité, CNRS UMR 6047, Unit for Integrated Mycobacterial Pathogenomics, 75015 Paris, France; Institut Pasteur, Université Paris Cité, CNRS UMR 2000, Unit for Human Evolutionary Genetics, 75015 Paris, France; Institut Pasteur, Université Paris Cité, CNRS UMR 6047, Unit for Integrated Mycobacterial Pathogenomics, 75015 Paris, France

**Keywords:** *Mycobacterium tuberculosis*, evolution, genetic diversity, population dynamics, virulence, host–pathogen relationship

## Abstract

Tuberculosis (TB) remains one of the deadliest infectious diseases in human history, prevailing even in the 21^st^ century. The causative agents of TB are represented by a group of closely related bacteria belonging to the *Mycobacterium tuberculosis* complex (MTBC), which can be subdivided into several lineages of human- and animal-adapted strains, thought to have shared a last common ancestor emerged by clonal expansion from a pool of recombinogenic *Mycobacterium canettii*-like tubercle bacilli. A better understanding of how MTBC populations evolved from less virulent mycobacteria may allow for discovering improved TB control strategies and future epidemiologic trends. In this review, we highlight new insights into the evolution of mycobacteria at the genus level, describing different milestones in the evolution of mycobacteria, with a focus on the genomic events that have likely enabled the emergence and the dominance of the MTBC. We also review the recent literature describing the various MTBC lineages and highlight their particularities and differences with a focus on host preferences and geographic distribution. Finally, we discuss on putative mechanisms driving the evolution of tubercle bacilli and mycobacteria in general, by taking the mycobacteria-specific distributive conjugal transfer as an example.

## Teaser

The causative agents of tuberculosis are represented by a group of closely related bacteria belonging to the *Mycobacterium tuberculosis* complex, which can be subdivided into several lineages of human-adapted and animal-adapted strains, thought to have shared a last common ancestor emerged by clonal expansion from a clade of recombinogenic *Mycobacterium canettii*-like tubercle bacilli.

## Introduction

Tuberculosis (TB) remains one of the key infectious diseases that has plagued humankind for thousands of years and continues to claim an enormous death toll in many vulnerable populations, even in the 21^st^ century. The latest WHO TB report mentions 10.6 million new cases and 1.3 million TB deaths in 2022 (World Health Organization 2023). Tuberculosis as a disease was already known in antiquity and described by Hippocrates under the name of phthisis, which means consumption. Also known as the white plague, because of the pallor of the patients, the disease is accompanied by lung pain, sweating, and severe weight loss. Without a cure, TB is often a death sentence. As such, TB has long been considered a hereditary disease. However, some exceptions to this belief were also formulated, such as the descriptions of Benjamin Marten, an English physician, who in 1720 hypothesized in his publication “*A new theory of consumptions”* that TB could be caused by “wonderfully minute living creatures,” which could be transmitted from consumptive patients to sound patients during close and prolonged contact (Marten 1720). Many years later in 1865, Jean-Antoine Villemin, a French physician, demonstrated the contagious and transmissible nature of TB by injecting tuberculous tissue from TB patients into rabbits, which then became infected and tuberculous (Villemin 1865). In this way, he anticipated the existence of a specific agent responsible for TB. The final demonstration of the bacterial origin of TB was done a few years later by the German physician Robert Koch in 1882 through the isolation of the etiological agent of TB, which he named *Mycobacterium tuberculosis* (Koch 1882). With several examples, which confirmed the germ theory that was initially proclaimed by Louis Pasteur, the end of the 19^th^ century became an outstanding era for the emergent discipline of microbiology (Jagielski 2023). Indeed, numerous major diseases such as anthrax, cholera, TB, or plague were then identified as infectious diseases, and the causative pathogens were isolated. The discovery of the infectious nature of TB opened completely new perspectives for the fight against this deadly disease, which still continues today. Despite the enormous knowledge that has been accumulated in >140 years of research since the discovery of the TB bacillus, many open questions remain, which is also due to the fact that TB is a multifactorial, highly complex disease that depends on various attributes of the pathogen, but also on the host and the environmental conditions in which the host lives. It is reported that “only” 5%–10% of all people who become infected with *M. tuberculosis* will develop acute, clinical TB disease in their lifetime, whereas the remaining 90%–95% of people will control the *M. tuberculosis* infection, leading to persistent, subclinical forms of the infection or in some cases, elimination of the pathogen (Behr et al. 2018, World Health Organization 2023). The different aspects of host–pathogen interaction are the subject of ongoing international research, whereby a better understanding of how *M. tuberculosis* can circumvent and rearrange the host response remain key research questions. This review is focused on the evolution and emergence of *M. tuberculosis* and related TB-causing mycobacteria, hereafter referred to as tubercle bacilli, whereby the main aim is to provide an overview as well as some selected examples of the likely reasons and factors that have contributed to the emergence and outstanding evolutionary “success” of *M. tuberculosis* as a human pathogen.

## The 25^th^ anniversary of the *M. tuberculosis* genome

A strong impact on deciphering the evolution of *M. tuberculosis* has come from comparative genomics, a discipline that emerged ~25 years ago with the publication of the first mycobacterial genome sequence derived from the widely used reference strain *M. tuberculosis* H37Rv (Cole et al. 1998). The original motivation for undertaking the whole genome sequencing project in an era when DNA sequencing technologies were still tedious, expensive, and time-consuming was to provide a new understanding of the genetics, biochemistry, physiology, and pathogenesis of the etiological agent of TB, and to complete this approach by functional and comparative genomics, which can provide new insights into the evolution of tubercle bacilli and related mycobacterial pathogens. The genome sequencing project revealed a genome size of 4411529 bp for the *M. tuberculosis* H37Rv genome and an average G + C content of 65.5%, whereby some genomic regions showed an exceptionally high G + C content of ~80%, which were found to correspond to the genomic regions encoding proteins of the so-called PE and PPE families, named after their characteristic Pro-Glu (PE) or Pro-Pro-Glu (PPE) motifs (Cole et al. 1998). In this initial analysis, 50 genes encoding stable RNA species and 3924 genes encoding proteins were identified, accounting for 91% of the potential coding capacity. More detailed analyses showed that 59% of the *M. tuberculosis* genes were predicted to be transcribed with the same polarity as the replication forks, which could be a reflection of its slow growth as it is a lower percentage than seen for other, faster-growing microorganisms, such as *Bacillus subtilis* (75%) that seem to obtain higher gene expression levels by coordinating directions of transcription and replication (Cole 1999). At the time of original publication, functions were confidently attributed to ~40% of the protein-coding genes and for another 40% of genes some information or similarity was found, although many of these genes belonged to the class known as conserved hypotheticals. Follow-up in silico analyses of the predicted *M. tuberculosis* proteome showed that over half of the proteins resulted from ancestral gene duplication or domain shuffling events, while one-sixth showed no similarity to polypeptides described in other organisms. Prominent among the genes that appeared to have been duplicated on numerous occasions were those involved in fatty acid metabolism, regulation of gene expression, and those encoding the unusually glycine-rich PE and PPE proteins (Tekaia et al. 1999). Further protein similarity analyses, coupled with inspection of the genetic neighborhood of genes uncovered several new gene families that apparently were the results of gene duplication and modification events that occurred throughout the evolution of *M. tuberculosis* (Tekaia et al. 1999). Noteworthy among these is the 12-membered *mmpL* gene family encoding mycobacterial membrane proteins involved primarily in lipid transport functions. Another example is the *mce* gene family, which is constituting four *mce* operons in *M. tuberculosis*, some of which have later been shown to be involved in virulence and mycobacterial growth using cholesterol as substrate (Griffin et al. 2011). Finally, this analysis also revealed the existence of five *esx* operons in *M. tuberculosis*, which show similar genomic organization, and which have been later defined as the operons encoding a new type of bacterial secretion systems, named ESX or type VII secretion systems (Tekaia et al. 1999, Bitter et al. 2009, Gröschel et al. 2016). In this review, we will describe some of these gene families in more detail and will also present certain aspects of their evolution within the mycobacterial genus. In numerous functional studies in the last 25 years, new functions were attributed to many of the genes of unknown function and/or conserved hypotheticals, and with steadily improving sequencing technologies and analysis tools, the H37Rv reference strain was recently also re-sequenced and re-analyzed (Chitale et al. 2022). This analysis showed a genome size of *M. tuberculosis* H37Rv of 4417942 bp, which slightly differed from the previously determined H37Rv reference sequence by the presence of additional 6.4 kb of sequence, corresponding mainly to repetitive DNA encoding an IS*6110* transposase, insertions in PE/PPE genes, and new paralogs of *esxN* and *esxJ* genes that were apparently omitted in the original genome assembly of 1998 (Chitale et al. 2022).

The H37Rv genome sequence was also the basis for many postgenomic studies, such as genome-wide transposon insertion screens that searched for essential genes of *M. tuberculosis* during *in vitro* growth conditions (Sassetti et al. 2003, Griffin et al. 2011, Rock et al. 2017) or genes that were essential for the survival of *M. tuberculosis* during infection conditions (Camacho et al. 1999, Sassetti and Rubin 2003, Zhang et al. 2013). These studies initially revealed that ~600 genes of the genome of *M. tuberculosis* were essential for optimal *in vitro* growth and that ~200 genes were essential for the survival of *M. tuberculosis* under *in vivo* infection conditions. Over time, some variation in the number and identity of essential genes was noticed among different studies, which was likely due to the use of different models and growth media (Griffin et al. 2011; https://www.mtbtndb.app/analyze_datasets). However, a large overlap among the identified genes in the different studies was still noticed, which provides a high level of confidence for the phenotype of these genes.

As other important postgenomic approaches that directly used the H37Rv genome sequence, transcriptomic studies should be mentioned here. Indeed, the analysis of genome-wide patterns of gene expression in *M. tuberculosis* strains and selected mutants has strongly enriched the knowledge about the pathogen and its physiological conditions (Schnappinger et al. 2003, Boshoff et al. 2004, Homolka et al. 2010, Bosch et al. 2021, Bei et al. 2023). As one well-known example, the two-component regulon PhoP-PhoR (PhoPR) has attracted much attention. In various transcriptomic studies, PhoPR was shown to regulate >80 genes of *M. tuberculosis*, including many that are implicated in virulence (Walters et al. 2006, Solans et al. 2014). Interestingly, PhoPR was also shown to play an important role in the evolution of *M. tuberculosis* and related mycobacteria (Gonzalo-Asensio et al. 2014, Chiner-Oms et al. 2019), a subject that will be discussed in a separate paragraph, further below.

Taken together, it is clear that the first mycobacterial genome sequence was a game changer for many disciplines in mycobacterial research, including research on the phylogeny and evolution of mycobacteria. Today, genome sequencing has become a routine technology and thousands of mycobacterial genome sequences can be found in different databases. Besides those from many clinical isolates of *M. tuberculosis*, genome sequence databases also harbor a large variety of sequences from other mycobacterial species, which can provide new information on the phylogenetic inter- and intraspecies relationships in the genus mycobacteria.

### Evolution at the genus level

In the tree of life, the genus *Mycobacterium* represents a single entity within the family *Mycobacteriaceae*, which is part of the order *Mycobacteriales* with the phylum *Actinobacteria* (Magee and Ward 2012 et al. 2012, Hug et al. 2016) and comprises almost 200 named species of bacteria, most of them representing free-living, environmental species, and some that can cause infections of different degrees of severity in humans and animals. Identification of mycobacterial species is often based on the analysis of the 16S rRNA, and 16S–23S spacer sequences; and sequences from housekeeping genes such as *hsp65, rpoB*, and *gyrA* (Forbes et al. 2018, Gupta et al. 2018). However, it is also clear that quite a large genomic diversity exists among the mycobacterial species, which was used as an argument by Gupta and colleagues to propose a new classification scheme of the genus *Mycobacterium*, splitting the genus into five new genera named *Mycolicibacterium, Mycolicibacter, Mycolicibacillus, Mycobacteroides*, and an emended species *Mycobacterium* (Gupta et al.^2018^). While the species names linked to this proposed reclassification were rapidly included in a list of new names released in the International Journal of Systematic and Evolutionary Microbiology (Oren and Garrity 2018), and hence taken into consideration by certain large strain collections and databases such as the American Type Culture Collection (ATCC) and the National Center for Biotechnology Information (NCBI), the changes were strongly contested in large parts of the scientific community working on medically important mycobacteria. Indeed, a consortium of specialists in mycobacterial taxonomy suggested to ignore the new names (Tortoli et al. 2019) as these names were causing possible confusion in clinical treatment. In addition, an in-depth study to define the mycobacterial genus boundaries, using analyses of 16S rRNA gene similarities, amino acid identity indexes, average nucleotide identities, alignment fractions, and percentages of conserved proteins, revealed that the original *Mycobacterium* genus definition was better supported by the data obtained than the proposed split of the genus into five new genera (Armstrong and Parrish 2021, Meehan et al. 2021). Similar conclusions, except for one difference, were also drawn from a recent taxonomic study that used normalized tree clustering and network analysis of several genomic relatedness indices to establish taxonomic relationships among species belonging to the order *Mycobacteriales*. The difference noted was that this analysis supported the separation of the species within the new genus *Mycobacteroides* as taxonomically justified, whereas it considered the split into the other new genera as non-justified (Val-Calvo and Vázquez-Boland 2023). In light of all these arguments and uncertainties and since according to taxonomic rules novel and previous nomenclatures coexist and are synonyms (Tortoli et al. 2019), we prefer to use the traditional single genus mycobacterial nomenclature in this review article. For similar reasons, we will also use the traditional nomenclature for the names of the different species or ecotypes of the tubercle bacilli (Smith et al. 2006, 2009), which contrasts with the recent suggestion by Riojas and coworkers to rename all these different tubercle bacilli adapted to different hosts as *M. tuberculosis* (Riojas et al. 2018), a name that we would like to keep exclusively for the human-adapted pathogen to avoid confusion. We understand that the tubercle bacilli comprising the members of the *Mycobacterium tuberculosis* complex (MTBC) and those of a taxon named “Mycobacterium canetti” (van Soolingen et al. 1997) or *Mycobacterium canettii* (Pfyffer et al. 1998) share 98%–99.9% of genome sequence identity among each other (Smith et al. 2009, Supply et al. 2013) and therefore belong theoretically to a single bacterial species, but as they show clear differences in their host preferences, the traditional nomenclature of members of the MTBC will be used here. Likewise, despite the close genomic relationship between strains of the *M. canettii* taxon and the members of the MTBC, we consider the *M. canettii* strains as an outgroup that is not part of the MTBC. The reasons for this distinction are linked to the many differences that can be observed between *M. canettii* strains and MTBC members. Indeed, *M. canettii* strains are rare clinical isolates obtained from patients in the region of the Horn of Africa who may show various infection symptoms, ranging from lymph node and skin infections to active pulmonary TB (Fabre et al. 2004, Koeck et al. 2011, Supply et al. 2013). One of the most obvious differences between *M. canettii* and MTBC isolates is shown by the smooth colony morphology of *M. canettii* strains on solid culture media that contrasts with the rough colony morphology seen for members of the MTBC. The molecular mechanisms that underlie these differences were deciphered, showing that smooth *M. canettii* strains produce lipo-oligosaccharides (LOS), whereas this ability was lost during the evolution of the MTBC, likely through the recombination of two *pks5* genes and deletion of the intermediate *pap* gene (Boritsch et al. 2016a). *M. canettii* strains are also characterized by somewhat larger genomes and a considerably higher number of SNPs, which were shown to range between 16000 and 61000 relative to the reference strain *M. tuberculosis* H37Rv, compared to the MTBC that show ≤2400 SNPs between its members (Garnier et al. 2003, Supply et al. 2013, Blouin et al. 2014). However, in a recent report, an *M. canettii* clinical isolate from Ethiopia was described that is phylogenetically more closely related to the MTBC clade than to the previously reported *M. canettii* clade. This strain named ET1291 shares the characteristic smooth colony morphology and twin-*pks5* configuration of *M. canettii* strains but is separated by ~6000 SNPs from the reconstructed ancestral genome of the MTBC, and ~9000 SNPs from the closest, previously described *M. canettii* strain (Yenew et al. 2023). The example of this recent isolate shows that with the addition of new genomes, the phylogeny of tubercle bacilli will continue to be constantly refined, even though the global picture on the population structure of the tubercle bacilli seems to remain essentially the same. Finally, members of the *M. canettii* clade also show a recombinogenic population structure with frequent loci that were likely generated by horizontal gene transfer (HGT) events, which contrasts with the clonal population structure observed for MTBC members, a feature that will be discussed further below in a separate section on the specific type of HGT found in mycobacteria.

**Figure 1.**
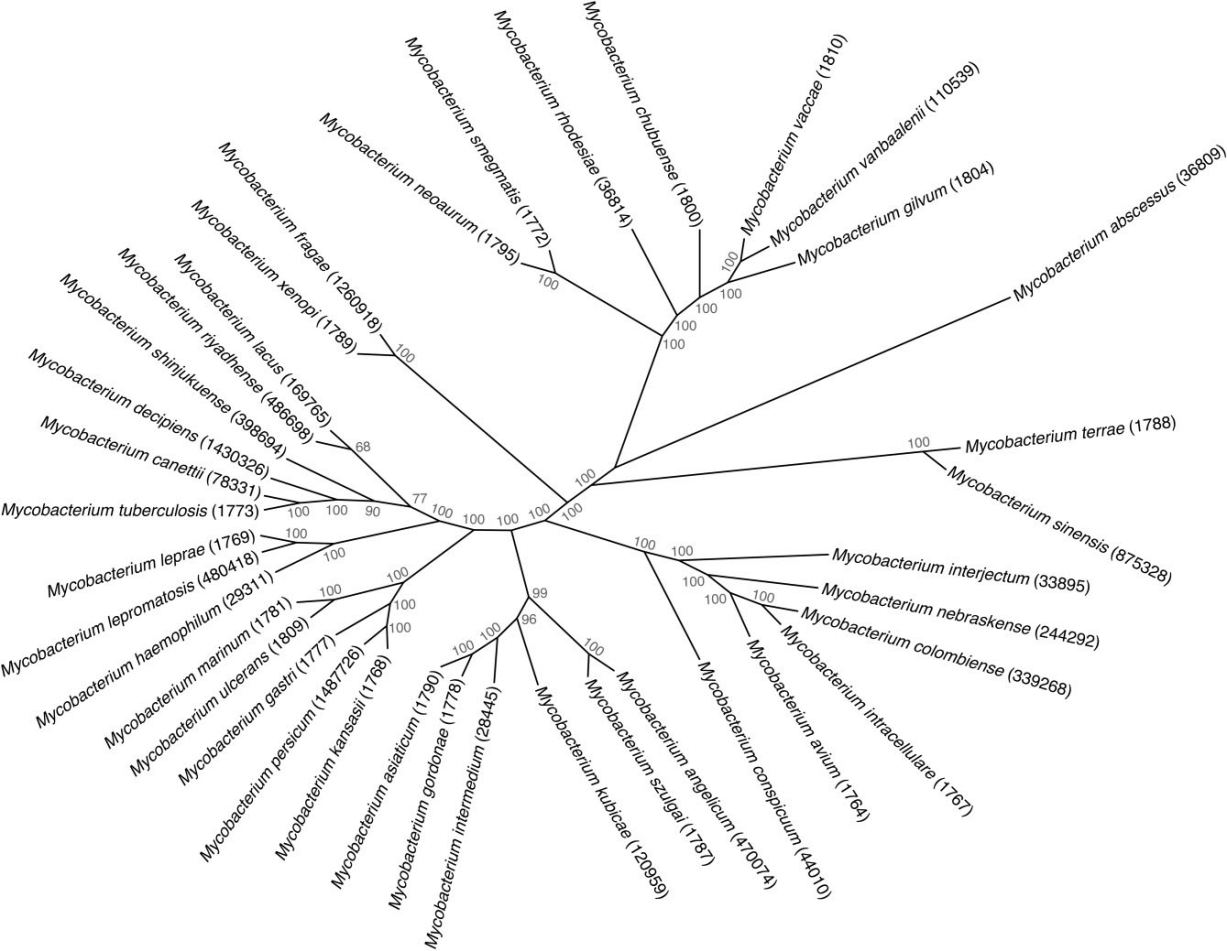
Phylogenetic topology of mycobacteria. Genomes of selected *Mycobacterium* genus strains were downloaded from the GenBank database and analyzed using PanACoTA v1.4.0 (Perrin and Rocha 2021). Genomes were annotated using Prokka v1.14.5 (Seemann 2014) and their pan-genome was inferred using MMseqs2 v14-7e284 (Steinegger and Söding 2017) based on a minimum sequence identity of 80% at the protein level. Genes conserved across all selected mycobacteria were aligned using MAFFT v7.522 (Katoh and Standley 2013). Maximum-likelihood phylogenetic reconstruction was performed using RAxML-NG v1.2.0 (Kozlov et al. 2019) with the generalized time reversible (GTR) substitution model, mean GAMMA distribution of rate heterogeneity with four categories (G), a maximum-likelihood estimate of stationary frequencies (FO), and 1000 bootstrap replicates. Bipartition support of the best-scoring tree rooted using *M. abscessus* was computed using the transfer bootstrap expectation metric from BOOSTER v0.1.2 (Lemoine et al. 2018). The resulting maximum-likelihood phylogenetic tree was drawn as a cladogram with the daylight layout and no branch length scaling using the R package ggtree v3.6.2 (Yu et al. 2017). Taxonomy IDs of selected mycobacteria are indicated in brackets and bootstrap support values are depicted in gray as percentages.

In any case, the increasing number of available mycobacterial genome sequences allowed the phylogenetic relationships of a wide range of mycobacterial species to be evaluated, including those considered as being closely related to *M. tuberculosis* (Fig. 1). Previous comparative genomic approaches, using *Mycobacterium marinum* or *Mycobacterium kansasii* as closest related outgroups known at the time (Stinear et al. 2008, Wang et al. 2015) suggested that the emergence of *M. tuberculosis* as a key human pathogen from environmental non-tuberculous mycobacteria (NTM) was accompanied with important genomic changes, such as gene gain by HGT, gene duplication and diversification events as well as massive gene loss, resulting in genome size reduction and adaptation to new environments and hosts. These genome-wide analyses also revealed a wide evolutionary gap between the known environmental mycobacteria and the tubercle bacilli, suggesting the existence of yet unknown evolutionary intermediates (Veyrier et al. 2011, Wang and Behr 2014, Sapriel and Brosch 2019). The identification and description of several new mycobacterial species in the last 25 years that appeared genomically more closely related to *M. tuberculosis* than the previously used comparator species *M. marinum* and/or *M. kansasii* (Tortoli et al. 2017) confirmed that such intermediates existed. The four concerned mycobacterial species were isolated from clinical samples in different parts of the world and were named *Mycobacterium decipiens, Mycobacterium lacus, Mycobacterium riyadhense*, and *Mycobacterium shinjukuense* (Fig. 1).

*Mycobacterium decipiens* was isolated in 2012 and in 2016 from a 58-year-old woman and a 5-year-old girl, respectively (Simner et al. 2014, Brown-Elliott et al. 2018). In both patients, symptoms appeared after their holidays in a tropical area that were associated with swelling and pain in the thumb and wrist for the first case, and in the abdomen with fever for the second case. Mycobacterial strains were isolated and identified as non-specified *Mycobacterium* showing mycolic acid profiles in high-performance liquid chromatography (HPLC) analyses that were close to those of *M. tuberculosis*. Moreover, 16S rRNA sequences showed 99.4% similarity with those of the members of the MTBC, but the average nucleotide identity (ANI) value indicating the nucleotide-level genomic similarity between the coding regions of the genomes of the isolates and those of members of MTBC was below 98%, suggesting that the isolates were not belonging to the MTBC. The isolates were described as belonging to a new species of slow-growing mycobacteria, named *M. decipiens* (Simner et al. 2014, Brown-Elliott et al. 2018).

*Mycobacterium lacus* was isolated in the year 2000 from a bursitis of the elbow of a 68-year-old woman (Turenne et al. 2002). It was hypothesized that the infection happened during a minor elbow injury in a lake in Canada, even though there was no wound observed. The elbow became painful and swollen and a medical intervention was made to excise the bursitis. Six months thereafter the bursitis was still there and mycobacteriological tests revealed the presence of acid-fast bacteria that were identified as a new slow-growing mycobacterial species named *M. lacus*, for which it was the only case described up to now (Turenne et al. 2002).

*Mycobacterium riyadhense* was first isolated from a 19-year-old patient in 2009 in Saudi Arabia. The patient suffered from eye pain after a blunt trauma. Bacterial analysis of the sinus lavage identified slow-growing mycobacteria of a new species that was named *M. riyadhense* (van Ingen et al. 2009). Since this first isolation, 24 additional cases of *M. riyadhense* infections were described, including pulmonary, bone, spine, brain, and lymph node infections (van Ingen et al. 2009, Choi et al. 2012, Godreuil et al. 2012, Saad et al. 2015, Varghese et al. 2017, Alenazi et al. 2019, Guan et al. 2021).

*Mycobacterium shinjukuense* was isolated from pulmonary infections that occurred in Japan between 2004 and 2006 in immunocompetent patients who were 57–89 years old (Saito et al. 2011). Slow-growing mycobacteria were isolated and characterized, showing 97.8% sequence similarity of their 16S rRNA with that of MTBC members. The new species was named *M. shinjukuense* (Saito et al. 2011, Takeda et al. 2016). Since 2004, >10 cases of *M. shinjukuense* lung infections were described but their number might have been underestimated due to potential confusion with *M. tuberculosis* infections (Taoka et al. 2020).

**Figure 2.**
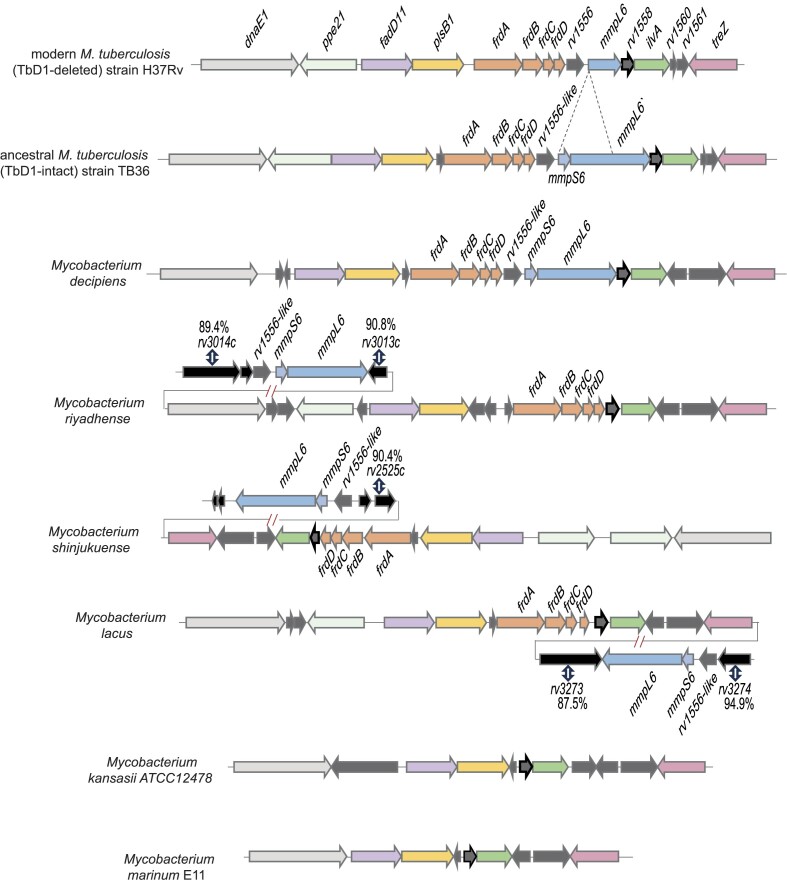
Genomic representation of the fumarate reductase locus in modern (TbD1-deleted) and ancestral (TbD1-intact) *M. tuberculosis* strains, *M. decipiens* (ATCC TSD-117), *M. lacus* (DSM 44577), *M. riyadhense* (DSM 45176), *M. shinjukuense* (DSM 45663), *M. kansasii* (ATCC 12478), and *M. marinum* (E11). Comparisons were performed using the Artemis Comparison Tool (Carver et al. 2005) and the MicroScope database (Vallenet et al. 2017). Genes surrounding the genomic locus containing the orthologues of *rv1556, mmpL6*, and *mmpS6* in *M. lacus, M. riyadhense*, and *M. shinjukuense* were compared to *the M. tuberculosis* H37Rv genome and percentages of amino acid identities with *M. tuberculosis* genes determined by the MaGe tool (Vallenet et al. 2006) are indicated.

The isolation and initial genomic characterization of these four new species opened new possibilities for comparative evolutionary analyses (Sapriel and Brosch 2019). The use of dedicated phylogenomic approaches for the comparison of the four species with selected other mycobacterial species, including the two previous comparator species *M. kansasii* and *M. marinum* as well as *M. tuberculosis* and *M. canettii*, showed that the four species formed a new clade together with the tubercle bacilli, named *M. tuberculosis*-associated phylotype. In good accordance with these results, the study also revealed that *M. tuberculosis* shared higher ANI values with the four new mycobacterial species (80%–85%) than with *M. marinum* (78%) and *M. kansasii* (79%), and the existence of the common clade including the four mycobacterial species and tubercle bacilli was also seen in phylogenetic analyses based on >100 universally conserved bacterial genes (Sapriel and Brosch 2019). This study also focused on genes encoding selected virulence factors that are thought to have been acquired by HGT during the speciation of the tubercle bacilli. The results of this analysis showed that 35 genes encoding proteins involved in survival or growth of *M. tuberculosis* during infection of mononuclear phagocytic cells or in animal models were shared in some or all the species of the *M. tuberculosis*-associated phylotype, revealing that some virulence characteristics of the tubercle bacilli that were previously considered as being exclusively present in tubercle bacilli, had likely been acquired before the speciation of *M. tuberculosis* by a common ancestor shared with *M. decipiens, M. lacus, M. riyadhense*, and *M. shinjukuense*. As an example, Fig. 2 shows a graphical representation of the genes of the fumarate reductase locus that is present in all *M. tuberculosis*-associated phylotype species but absent from *M. kansasii* and all other known mycobacterial species outside the *M. tuberculosis*-associated phylotype.

This locus is a particularly interesting region as it contains (*i*) the genes *frdA, frdB, frdC*, and *frdD* encoding a fumarate reductase; and (*ii*) the genes encoding a transcriptional regulator, MmpS6 and MmpL6 representing a putative transmembrane transporter that belong to the MmpS/MmpL membrane protein family. Fumarate reductase is a membrane-bound bifunctional enzyme needed for maintaining the mycobacterial membrane in an energized state under anaerobic conditions (Watanabe et al. 2011). One could speculate that the acquisition of the fumarate reductase locus might have enabled the members of the *M. tuberculosis*-associated phylotype to better adapt to survival and growth under anaerobic conditions, providing the recipients of the HGT with a potential evolutionary advantage during the transition from environmental bacteria to host-adapted pathogens. However, next to the *frdA, frdB, frdC*, and *frdD* genes, the second part of the locus is composed of genes encoding a transcriptional regulator and the MmpS/L6 transporter. Interestingly, the genes of this putative genomic island are present in all *M. tuberculosis*-associated phylotype members. However, the synteny of the genes is conserved only in tubercle bacilli and *M. decipiens*, since they are divided into two separate loci in *M. lacus, M. riyadense* and *M. shinjukuense* (Fig. 2). The function of the second part composed of the genes encoding the transcriptional regulator and the MmpS/L6 transporter remain for the moment unknown, especially as the epidemiologically most widely distributed and abundant lineages of the MTBC have deleted the *mmpS6* gene and parts of the *mmpL6* gene at a later stage of evolution. Indeed, this deletion of the *M. tuberculosis*-specific deletion region 1 (TbD1) characterizes all *M. tuberculosis* strains of lineages 2, 3, and 4 (L2, L3, and L4) and was first identified in 2002 in a study that investigated large sequence polymorphisms within a representative sample collection of MTBC strains (Brosch et al. 2002), as described below in more detail in a paragraph dedicated to the evolution of different strain lineages within the MTBC. The fumarate reductase core locus represents a striking example of a genomic region that was likely acquired by HGT by the last common ancestor of the *M. tuberculosis*-associated phylotype members. However, there exist several other examples of genomic loci that are shared only by *M. decipiens* and the tubercle bacilli, such as the sulpholipid synthesis locus, the fucosyltransferase locus, the *mymA* operon representing the VirS virulence regulation locus (*Rv3082c* and *virS*), or the LipF lipase-esterase encoding region (Sapriel and Brosch 2019), some of which were previously defined as tubercle bacilli-specific regions on the basis of comparisons with *M. marinum* (Stinear et al. 2008). These examples emphasize that *M. decipiens* is the most closely related mycobacterial species to the tubercle bacilli, currently known, which is also reflected in the high degree of protein similarity for most of its proteome (Sapriel and Brosch 2019). However, despite the many gene orthologues shared between *M. decipiens* and the tubercle bacilli, there remain some regions that seem to be specific to tubercle bacilli only. For example, genes of a lipid glycosylation locus (*rv0112*–*rv0115*, named *gca, gmhA, gmhB*, and *hddA*) predicted to encode enzymes involved in lipid modification are exclusively found in tubercle bacilli based on the currently available mycobacterial genomes in public databases and have also been previously defined as part of a genomic island (Becq et al. 2007).

The evolution of tubercle bacilli at the genus level was not only strongly shaped by gene gain via HGT, but also by gene loss. As demonstrated very clearly by the dramatic gene loss and gene decay observed in the 3.2-Mb-sized *Mycobacterium leprae* genome (Cole et al. 2001), reductive evolution events may also contribute to the adaptation and specialization of mycobacteria to specific environments. Likewise, it is thought that during its long-term evolution, the genome size of *M. tuberculosis* was reduced to 4.4 Mb, compared with 6.4 Mb and 6.6 Mb for the environmental mycobacterial species *M. kansasii* and *M. marinum*, respectively (Veyrier et al. 2011, Sapriel and Brosch 2019). Even when looking at the members of the *M. tuberculosis*-associated phylotype, it can be observed that the genome size of *M. riyadhense* resembles that of *M. kansasii* and *M. marinum* with 6.2 Mb, whereas *M. decipiens* shows a smaller genome of 5.3 Mb similar to the one of *M. lacus* (5.1 Mb). Only *M. shinjukuense* exhibits a 4.5 Mb genome size that is similar in size to that of *M. tuberculosis*. Among the tubercle bacilli, certain *M. canettii* strains show slightly larger genome sizes than *M. tuberculosis* of ~4.5 Mb (Supply et al. 2013, Sapriel and Brosch 2019). Taken together, the analysis of genome sizes of selected mycobacterial species gives some indication of the probable evolutionary pathway from environmental mycobacteria carrying large genomes towards opportunistic and obligate pathogens with smaller genomes, although there is no clear correlation with the genomic proximity at the nucleotide and amino acid levels, as seen with the close genomic similarity but different genome sizes for *M. decipiens* and the tubercle bacilli.

### Evolution of tubercle bacilli

**Figure 3.**
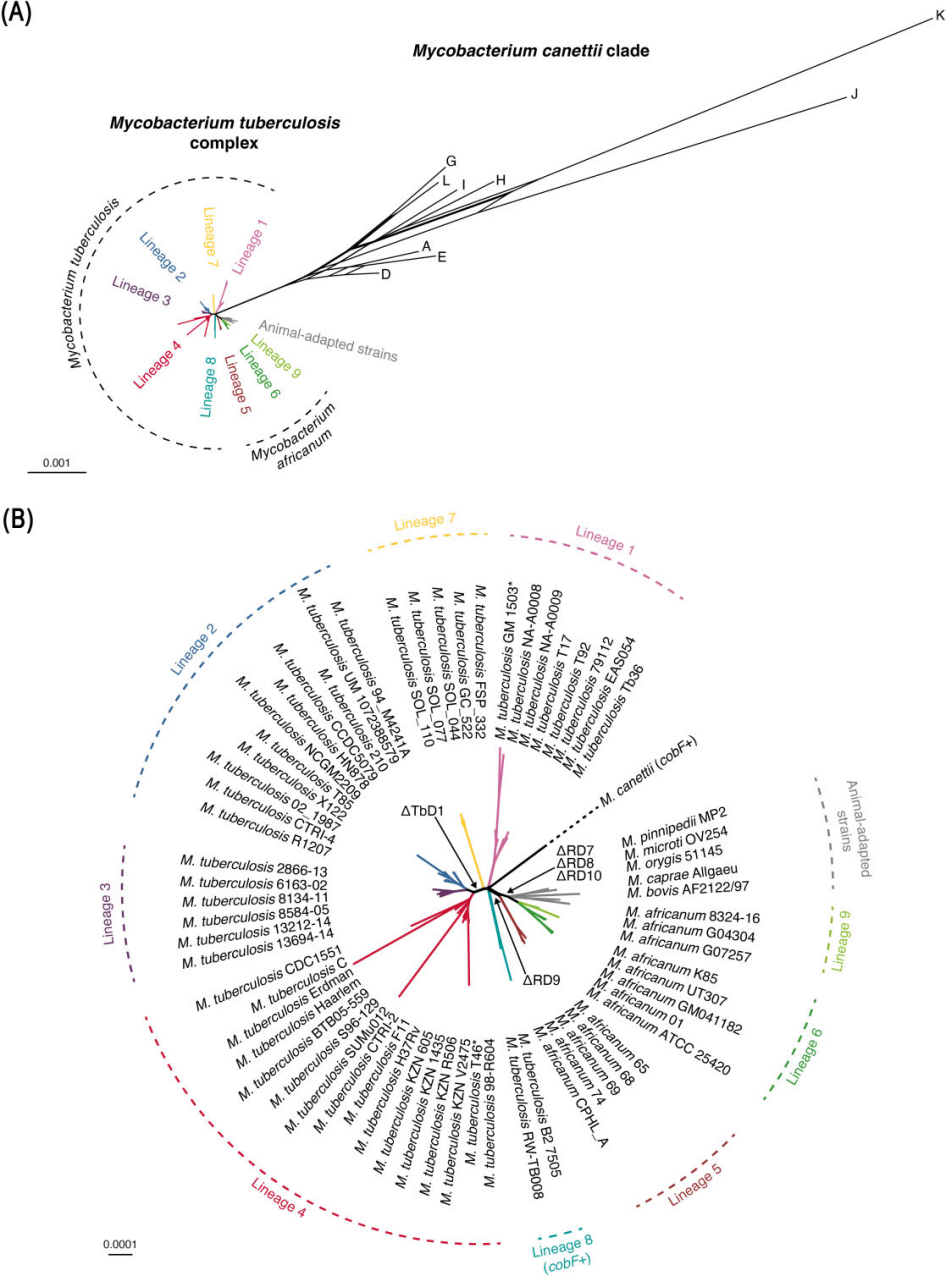
(A) Phylogenetic topology of *M. canettii* strains and members of the MTBC. Genomes of selected *M. canettii* and MTBC strains were downloaded from the GenBank database and analyzed using PanACoTA v1.4.0 (Perrin and Rocha 2021). Genomes were annotated using Prokka v1.14.5 (Seemann 2014) and their pan-genome was inferred using MMseqs2 v14-7e284 (Steinegger and Söding 2017) based on a minimum sequence identity of 95% at the protein level. Genes conserved across all selected mycobacteria were aligned using MAFFT v7.522 (Katoh and Standley 2013). Neighbor-Net network was computed from pairwise distances estimated with the Jukes and Cantor substitution model (JC69) using the R package phangorn v2.11.1 (Schliep 2011). The resulting unrooted phylogenetic network was drawn using the R packages tanggle v1.4.0 and ggtree v3.6.2 (Yu et al. 2017). (B) Higher magnification of the Neighbor-Net topology of the MTBC from (A). Genomic loss events such as the deletion of RD7-RD8-RD10, RD9, and TbD1 regions are indicated by arrows. The presence of the *cobF* gene in *M. canettii* and L8 genomes is indicated as “*cobF*+”, all other depicted genomes are *cobF*-deleted. Scale bars represent the number of substitutions per site. NB: We note a discrepancy in the MTBC phylogeny regarding both *M. tuberculosis* strains T46 and GM 1503, which were originally classified as belonging to the L1 and L4 lineages, respectively, based on selected genes (Hershberg et al. 2008) and whole-genome (GenBank: ACHO00000000.1; ABQG00000000.1) sequencing. More recently released genome sequences for T46 and GM 1503 strains (GenBank: JLCS00000000.1; JLCR00000000.1) were used here and the present topology depicts GM 1503 as part of the lineage L1 and T46 as part of the lineage L4 (denoted with *), which is consistent with the presence of an intact and a deleted TbD1 region in their genome sequence, respectively. We assume that the newer genome versions have been mislabeled in the database and that the original classification of T46 and GM 1503 within the lineages L1 and L4, respectively, is correct.

Tubercle bacilli are mycobacteria that can cause TB or TB-like disease in mammalian species, whereby the large majority of strains belongs to the MTBC, a few strains belong to the *M. canettii* clade, which can be considered as a closely related outgroup of the MTBC that is thought to resemble in many characteristics the putative ancestor of the MTBC (Supply et al. 2013, Tientcheu et al. 2017). As mentioned above, genomic and phenotypic comparisons of MTBC members and *M. canettii* strains have shown that *M. canettii* strains display greater genomic variability and many inter-strain recombination traces, compared to the clonal population of MTBC strains (Supply et al. 2013). For *M. canettii*, only a few cases of human TB have been reported, which are commonly linked to the geographical area of the Horn of Africa. This shows that *M. canettii* strains can cause human infections, but no human-to-human transmission was observed (Blouin et al. 2014). It was also reported that *M. canettii* strains were less demanding on specific growth media, as shown by their growth on trypticase-soy media and a shorter generation time in liquid medium, which might be advantageous features in environmental reservoirs (Koeck et al. 2011), although all known *M. canettii* strains correspond to patient isolates and no isolation of *M. canettii* from environmental sources was yet achieved (Supply and Brosch 2017, Gagneux 2018). However, the recombinogenic genome structures and above-mentioned phenotypic characteristics make a hypothetical environmental link of *M. canettii* strains plausible, even though the absence of pigmentation in *M. canettii* strains differs from typical environmental mycobacteria, such as *M. marinum, M. kansasii*, or *Mycobacterium avium*. These waterborne mycobacterial species produce a yellow pigment, which is thought to protect them from ultraviolet radiation, oxidants, and other environmental causes of damage. This faculty is due to the presence of a *crtEIB* cluster in their genomes, responsible for carotenoid biosynthesis involved in photochromogenicity (Ramakrishnan et al. 1997). The absence of these genes from *M. canettii* and MTBC strains, as well as from the other members of the *M. tuberculosis*-associated phylotype (*M. decipiens, M. lacus, M. riyadhense*, and *M. shinjukense*) suggests that the putative common ancestor of this group of mycobacteria might not have shared the same environmental niche as the waterborne NTM species *M. marinum, M. kansasii*, or *M. avium*. However, even if the hypothetical environmental niche of *M. canettii* strains remains currently unknown, the very close genomic relatedness between *M. canettii* and the MTBC, combined with the recombinogenic population structure and the different epidemiological situation render *M. canettii* strains of great value for evolutionary investigations that help to explain the evolutionary pathway from *M. canettii*-like ancestors towards the MTBC, finally leading to the obligate human pathogen *M. tuberculosis* (Fig. 3A).

An experimental *in vivo* evolution approach in mice was recently performed with two *M. canettii* strains, more or less distantly related to *M. tuberculosis* (Allen et al. 2021). By this approach, *M. canettii* mutants were obtained that appeared to have an enhanced *in vivo* persistence and to be also more resistant than their parental strains to nitric oxide. Genome sequencing of these mutants revealed mutations in two genomic loci: (*i*) one encoding an orthologue of Rv1339, an *M. tuberculosis* H37Rv protein that corresponds to a phosphodiesterase degrading cyclic-AMP (cAMP) (Thomson et al. 2022); and (*ii*) one encoding PE and PPE proteins of the ESX-5 type VII secretion system. Overall, the findings of this experimental evolution study in mice mirrored the natural evolution of *M. tuberculosis*, which is characterized by the increasing gain of the ability to withstand host-induced stresses and to persist inside the mammalian host (Supply et al. 2013, Allen et al. 2021). However, it is clear from various studies that the evolution of tubercle bacilli towards increased persistence and virulence is a multifunctional process and implies sophisticated regulatory networks. One such important virulence regulator system in tubercle bacilli is the PhoPR two-component signal transduction system, which is composed of the sensor kinase PhoR and the response regulator PhoP. Such two-component systems play a major role in bacterial responses to changing environments including inside the host (Miller et al. 1989). In *M. tuberculosis* PhoPR is required for full virulence (Pérez et al. 2001) and controls either directly or indirectly >80 genes (Walters et al. 2006, Solans et al. 2014).

In a recent study, it was shown that natural mutations in the sensor kinase PhoR of *M. canettii* relative to *M. tuberculosis* strains impact the expression of the PhoP regulon and the virulence of the strains, whereby PhoP-controlled genes are expressed at lower levels in most *M. canettii* strains than in *M. tuberculosis* (Malaga et al. 2023), a feature which correlates with their levels of virulence and persistence in mice (Supply et al. 2013). Together with the results of another study that identified phoR as the only gene in tubercle bacilli that was under positive selection in MTBC but not in *M. canettii* (Chiner-Oms et al. 2019), one can hypothesize that mutations in the PhoR sensor kinase and the downstream expression differences in the PhoP regulon have been under selection during the early spread of human TB and the emergence of the MTBC. Mutations in PhoPR in selected members of the MTBC are also likely to have played an important role in the evolution of the MTBC members as suggested by phoPR allele switching experiments between *M. tuberculosis* and *Mycobacterium bovis* that found PhoPR-regulated functions higher expressed in human-adapted *M. tuberculosis* strains than in animal-adapted *M. bovis* strains (Gonzalo-Asensio et al. 2014). All these examples suggest that during the evolution of tubercle bacilli, the MTBC has emerged from an *M. canettii*-like ancestor through various adaptation events that were linked to certain mutations and gene deletions (Boritsch et al. 2014, 2016a, Orgeur and Brosch 2018). Whether or not this evolution from *M. canettii*-like ancestors towards the MTBC has also included HGT events remains a scientifically challenging question that is discussed in the separate section on distributive conjugal transfer (DCT), further below.

### Evolution of the MTBC

As described above, it is highly likely that the MTBC emerged as a clonal complex starting from an *M. canettii*-like ancestor strain (Fig. 3A) that adapted to the mammalian host by gaining the ability to resist host defenses and by increasing virulence and persistence. We hypothesize that this process was caused or accompanied by successive genomic changes, such as the recombination of two *pks5* genes associated with the deletion of the *pap* gene resulting in deficiency of LOS production (Boritsch et al. 2016a), mutation of the *phoR* gene encoding the sensor kinase of the PhoPR two-component virulence regulatory system (Malaga et al. 2023) and other genomic changes that differentiate MTBC strains from *M. canettii* strains. One additional key event in the emergence of the MTBC seems to have been the deletion of the *cobF* gene locus, encoding a precorring-6a synthase that is a component of the cobalamin/vitamin B12 synthesis pathway. The *cobF* gene locus was present in all tested *M. canettii* strains (Supply et al. 2013, Blouin et al. 2014) and most NTM species, but was absent from the MTBC (Boritsch et al. 2014). The only currently known exception in the MTBC is represented by two recently characterized *M. tuberculosis* strains that were isolated from East-African TB patients from Rwanda and Uganda. These two *M. tuberculosis* strains displayed the typical rough colony morphotype of MTBC members and carried the *cobF* gene locus in the same orthologous genomic region as *M. canettii* strains (Ngabonziza et al. 2020). Interestingly, these two strains also showed an intact *pks8* gene (Ngabonziza et al. 2020), like seen for *M. canettii* strains, whereas this gene is split into a truncated *pks8* and *pks17* in all other members of the MTBC due to a frameshift mutation (Supply et al. 2013, Boritsch et al. 2014). These findings suggest that the two East-African *M. tuberculosis* strains, which were classified as being members of a separate lineage of the MTBC, named lineage 8 (L8) (Ngabonziza et al. 2020), represent the earliest branching clade of the MTBC, currently known. L8 was added to the other previously defined MTBC lineages consisting of (*i*) *M. tuberculosis* L1, L2, L3, L4, and L7 and *Mycobacterium africanum* L5 and L6 strains, all known to cause TB in humans; and (*ii*) animal-adapted MTBC members that affect various mammalian animal species (reviewed in Gagneux 2018 and Orgeur and Brosch 2018; Brites et al. 2018). Most recently, lineage L9, which harbors *M. africanum* strains that diverge to some extent from *M. africanum* L6 strains, was also added. Interestingly, *M. africanum* L9 strains, which share many genomic characteristics with L6 strains, were isolated from patients in East Africa, whereas *M. africanum* L5 and L6 strains are commonly found in patients from West Africa (Coscolla et al. 2021). Selected strains of these and other MTBC lineages are depicted in the genome sequence-based Neighbor-Net network in Fig. 3. The phylogenetic topology shows a central point where L8, L1, *M. africanum*, and animal-adapted strain lineages, and the remaining group of L7, L2, L3, and L4 branch from the *M. canettii* clade. Given the fact that L8 strains still have an intact *cobF* gene similar to *M. canettii* strains, whereas *cobF* is deleted in strains of all other MTBC lineages, it is tempting to speculate that the deletion of *cobF* occurred in a common ancestor of all MTBC lineages except L8, which has its phylogenetic position close to the central branching point (Fig. 3B). Given the extremely rare frequency of isolation of *cobF*-proficient *M. tuberculosis* L8 or *M. canettii* strains from TB patients, in comparison with the relatively high frequency of isolation of strains belonging to *cobF*-deleted MTBC lineages, the deletion of the *cobF* gene locus could have thus contributed to enhance the intracellular parasitic lifestyle of these MTBC lineages due to the need for sequestering vitamin B12 from the host. Therefore, MTBC lineages with a stronger dependence on host-supplied substrates than their ancestors might have started to adapt better to different mammalian hosts, including man. As such, the MTBC represents a clonal group of tubercle bacilli that is composed of 9 lineages of human-adapted tubercle bacilli and various animal-adapted tubercle bacilli, the latter branching next to *M. africanum* L6 strains and thus representing a subcomplex inside the MTBC (Fig. 3B).

The phylogenetic position of the animal-adapted strains inside the MTBC was first revealed by an analysis of the presence or absence of selected regions of difference (RDs) in a collection of different MTBC and *M. canettii* strains (Brosch et al. 2002). In this study, animal-adapted strains were found to belong to a clade of tubercle bacilli that had deleted the region RD9 and subsequently regions RD7, RD8, and RD10, similar to *M. africanum* L6 strains. Interestingly, these genomic regions were not deleted in *M. canettii* and *M. tuberculosis* strains, which argued firmly against an evolutionary descendance of human-adapted *M. tuberculosis* strains from animal-adapted *M. bovis* strains, a common hypothesis in the 1990s before the genome sequences of MTBC members were available (Stead et al. 1995). These findings were also confirmed by a study by Mostowy and coworkers, who reported a similar phylogeny for the MTBC (Mostowy et al. 2002). Since then, with the advancement of sequencing technologies and analysis of thousands of MTBC genomes, the phylogenic tree of animal-adapted MTBC members was constantly refined, giving rise to four main clades named A1–A4 (Brites et al. 2018). In the detailed phylogenetic tree of RD7-to-RD10-deleted MTBC members, clade A1 (*Mycobacterium mungi, Mycobacterium suricattae*, the dassie bacillus, and the chimpanzee bacillus) clusters with the human-adapted *M. africanum* lineages L6 and L9, whereas clade A2 (*Mycobacterium microti* and *Mycobacterium pinnipedii*), clade A3 (*Mycobacterium orygis*), and clade A4 (*Mycobacterium caprae* and *M. bovis*) represent subpopulations of RD7-to-RD10-deleted MTBC strains that have further diverged from L6 strains (Brites et al. 2018, Coscolla et al. 2021). The phylogenetic position of RD7-to-RD10-deleted MTBC members seems to be very special within the MTBC, as this subgroup comprises human-adapted (L6 and L9) and animal-adapted (A1–A4) strains, whereas all other lineages of the MTBC represent exclusively human-adapted tubercle bacilli. Among the various possibilities that could have favored the crossing of the species barrier and the jump into the animal host, which seems to have occurred at least twice according to phylogenetic relationship studies of A1–A4, L6, and L9 strains (Brites et al. 2018, Coscolla et al. 2021), the loss of the RD8 region might have been a particularly important genetic event. Indeed, it was reported that the deletion of the RD8 region, which comprises various binding sites for transcriptional regulators upstream of the ESX-1-associated *espACD* operon, allowed RD8-deleted strains to regain secretion of key virulence factors linked to the ESX-1 type VII secretion system independent of the PhoPR, Lsr2, and MprAB regulatory systems (Gonzalo-Asensio et al. 2014). Hence, on one hand, it is tempting to speculate that the regained robust ESX-1 functions in RD7-to-RD10-deleted MTBC strains might have created the conditions for successful infection of new mammalian hosts (animal-adapted strains) while maintaining a certain faculty to successfully infect humans (*M. africanum* L6 and L9 strains) (Orgeur and Brosch 2018). On the other hand, one can also observe that several animal-adapted strains, such as *M. mungi, M. suricattae*, and the dassie bacillus from clade A1 and *M. microti* from clade A2 show variably sized deletions in the core ESX-1 locus (Brites et al. 2018, Orgeur et al. 2021), suggesting that natural infection cycles in certain animal hosts might not need the prominent ESX-1 virulence functions that are essential for *M. tuberculosis* infection in humans.

Apart from changing the perspective on the phylogenetic position of animal-adapted MTBC strains, the knowledge on the distribution of RD regions in MTBC strains also allowed human-adapted MTBC members to be better differentiated (Brosch et al. 2002). Indeed, it was also found in this study that almost all *M. tuberculosis* strains investigated showed a deletion of the TbD1 region, characterized by deletion of the *mmpS6* gene and truncation of the adjacent *mmpL6* gene, whereas this genomic region was intact in *M. africanum* strains, animal-adapted strains, and a few *M. tuberculosis* strains of Southeast Asian origin. The presence or absence of the TbD1 region was then also used to define strains that had deleted this region (ΔTbD1) as “modern” *M. tuberculosis* strains, whereas TbD1-intact *M. tuberculosis* strains were defined as “ancestral” *M. tuberculosis* strains, as they resembled in that particular genomic locus the *M. canettii* strains, which show an intact TbD1 locus (Brosch et al. 2002). The presence or absence of the TbD1-region turned out to be a powerful marker for the differentiation of *M. tuberculosis* strains within the MTBC, whereby *M. tuberculosis* L2, L3, and L4 specifically represented “modern” ΔTbD1 *M. tuberculosis* strains, and all other lineages and clades of the MTBC represented “ancestral” TbD1-intact MTBC strains (Fig. 3B). Recently, it was found that the deletion of *mmpS6* and part of the *mmpL6* gene generated a fitness advantage for the strains under certain conditions of oxidative stress and during hypoxia (Bottai et al. 2020), suggesting that the deletion of the TbD1 region in a common ancestor of L2, L3, and L4 *M. tuberculosis* strains might have contributed to the wide distribution and global spread of strains belonging to these “modern” lineages that is particularly evident for L2 and L4 *M. tuberculosis* strains, which belong to the most frequently isolated *M. tuberculosis* strains worldwide.

Genomic differences between “ancestral” and “modern” lineages of *M. tuberculosis* were also attributed to unusually high rates of extrapulmonary dissemination and bone disease caused by an “ancestral” *M. tuberculosis* strain of L1 (Saelens et al. 2022). A closer inspection of the potential underlying molecular determinants for the observed differences identified EsxM, a secreted antigen of the ESX-5 type VII secretion system as a likely candidate for the differences. While EsxM was found intact in “ancestral” lineages, such as L1 and L5–L7, strains of “modern” lineages L2, L3, and L4 all harbored a truncated version of the *esxM* gene, consistent with a role for EsxM in regulating the extent of dissemination (Saelens et al. 2022). Altogether, these examples suggest that subtle genomic differences that have occurred during the evolution of the MTBC might have had an important impact on the infection potential of the concerned *M. tuberculosis* lineages in certain hosts, thereby impacting the interaction with different host populations, a subject that will be further discussed in the section below.

### Is there a co-evolution of MTBC with the host?

It has long been proposed that TB susceptibility is a function of both *M. tuberculosis* and host genetics. An early twin study in New York State has shown that 66.7% (52/78) of monozygotic (identical) twin siblings of TB cases have developed active disease, while this percentage for dizygotic (non-identical) twins was 23% (53/230) (Kallmann and Reisner 1947). More recently, candidate gene and GWAS studies have identified numerous loci implicated in TB susceptibility, but replicating the results of these studies has proven to be difficult, probably due to heterogeneity in phenotype definition and different epidemiological settings (reviewed in Naranbhai 2016 and Abel et al. 2018). Extensive studies of Mendelian susceptibility to mycobacterial disease (MSMD), a rare disease caused by single-gene inborn defects in interferon-γ (IFN-γ) immunity, have also helped pinpoint host genes that could play a role in developing TB in some patients (Boisson-Dupuis 2020). Inborn immune deficiencies, such as the one in tyrosine kinase 2 (TYK2), a Janus kinase associated with several cytokine receptors, have sparked particular interest in recent years. Namely, homozygosity for TYK2 variant P1104A was found to be enriched in a cohort of TB patients from several endemic regions (Boisson-Dupuis et al. 2018). The same study has shown that this variant appears to specifically impair IL-23-dependent IFN-γ induction. Consequently, a study of TYK2 P1104A in the UK Biobank cohort (Bycroft et al. 2018) has shown that 1% of TB patients of European ancestry are homozygotes (Kerner et al. 2019). Moreover, by analyzing ancient DNA (aDNA) from 1013 human genomes covering the period from the Mesolithic to the Middle Ages, it has been demonstrated that the frequency of TYK2 variant P1104A in Europe was much higher in the past, peaking at ~10% during the Bronze age. Starting from ~2000 years ago, there was a sharp decline in the frequency of this variant, coinciding with the high burden exerted by TB over the population of Europe, representing an example of negative selection under pathogen pressure (Boisson-Dupuis et al. 2018, Kerner et al. 2021).

As demonstrated by the example of purging of TYK2 P1104A from the European population, it is evident that *M. tuberculosis* may have played a role in shaping host genetics by exerting significant selective pressure over time. Similarly, we can expect that *M. tuberculosis* was subjected to selective forces in order to adapt to host populations and environments. As aforementioned, MTBC comprises nine lineages of human-adapted TB-causing mycobacteria, some of which are widespread and others are geographically restricted, sometimes also referred to as ecological generalists and specialists, respectively. While “ancestral” lineages could be considered specialists, the three evolutionary “modern” lineages (L2, L3, and L4) are generalists due to their distribution spanning continents (Gagneux 2012). However, L4, considered to be the most geographically widespread lineage is actually comprised of several sublineages. While three of them (L4.1.2/Haarlem, L4.3/LAM, and L4.10/PGG3) are found in almost 50 countries each, the sublineages L4.1.3/Ghana, L4.5, L4.6.1/Uganda, and L4.6.2/Cameroon are geographically restricted, generally to a few neighboring countries (Stucki et al. 2016). Even though the human T-cell epitopes were shown to be evolutionarily hyperconserved across *M. tuberculosis* lineages (Comas et al. 2010), the L4 generalists have more variable epitopes than the specialist sublineages, which could reflect the interaction of generalist strains with more diverse host populations (Stucki et al. 2016).

The hypothesis that *M. tuberculosis* lineages are adapted to specific human populations was also reinforced by the observation that different strains are usually transmitted to their sympatric hosts. Indeed, epidemiological studies in cosmopolitan centers such as San Francisco (Hirsh et al. 2004, Gagneux et al. 2006) and Montreal (Reed et al. 2009) indicate that a member of a particular population is preferentially infected by an *M. tuberculosis* strain that is associated with their region of origin. Moreover, in the case of HIV co-infection, this sympatric host–pathogen relationship is lost, and the patient is more likely to be infected with an allopatric strain (Fenner et al. 2013). However, it needs to be mentioned that there are social factors that could have also contributed to the preferential transmission of sympatric strains in these settings.

An example indicating local adaptation comes from a population genomics study of *M. tuberculosis* isolates from the Tibetan Plateau (Liu et al. 2021). The population of Tibet is relatively isolated, and there is a high burden of TB among highlanders (Jiang et al. 2023). Whole-genome sequencing of 567 *M. tuberculosis* isolates and subsequent analyses have shown that the so-called “modern Beijing” strains (L2.3), which are prevalent in the surrounding regions and worldwide, did not expand to Tibet and that the majority of Tibetan *M. tuberculosis* samples show signs of selection for truncating mutations in the *sseA* gene encoding a thiol-oxidoreductase (Liu et al. 2021). The authors hypothesize that *M. tuberculosis* was subjected to local selective pressures associated with oxidative stress, given the extreme living environment of the host population (Yang et al. 2017). Some authors propose that host–pathogen co-evolution eventually leads to less severe disease and that, on the contrary, a more recently formed lineage that has been introduced into a certain population leads to more severe disease, as the human population in question had not been historically exposed to it (Kodaman et al. 2014). This pattern seems to be particularly evident in the case of L4.6.1/Uganda when associated with an ancestral allele of the *SLC11A1* gene in individuals from Uganda. It is possible that L4.6.1/Uganda, being a recently derived sublineage, has not co-existed with the local population long enough for the individuals with the ancestral phenotype to produce an effective enough immune response (McHenry et al. 2020).

The notion of prolonged co-evolution between anatomically modern humans and MTBC has been supported by the confirmation of the African origin of both species—all existing MTBC lineages, including most animal-adapted strains infecting wild animals, as well as *M. canettii* can be found on the African continent (Hershberg et al. 2008, Comas et al. 2013). Efforts to reconstruct the evolutionary history of MTBC by using whole-genome sequencing data of extant strains have revealed that the genome-based phylogeny of the MTBC astonishingly resembles that of human mitochondrial genomes, indicating the co-divergence of the two species. In the same study, the MTBC was estimated to have emerged ~70000 years ago, long before the Neolithic demographic transition, which was marked by rapid growth of population, animal domestication, and adoption of agriculture (Comas et al. 2013). However, another work has used aDNA from pre-Columbian Peru to demonstrate that the most recent common ancestor (MRCA) of the MTBC has emerged ~6000 years ago (Bos et al. 2014). In addition, the authors have shown that the Peruvian ancient mycobacterial genomes did not cluster with human strains but rather resembled *M. pinnipedii*, also showing a pattern of deleted RD regions characteristic of the seal bacillus, thereby pointing to a putative pinniped-to-human zoonotic transfer (Bos et al. 2014). More recent research revealed that also individuals from precolonial populations with minimal access to marine resources seem to have been infected with *M. pinnipedii*-like strains, suggesting that in precolonial Americas, zoonotic transfer due to seal consumption in the coastal areas might have led to forms of human-adapted *M. pinnipedii*-like strains that were then spread further inland. Since *M. pinnipedii* is a member of an animal-adapted clade that diverged from a predominantly human pathogen (L5, L6, and L9 strains), such occurrence might represent an example of an animal-associated MTBC strain-type re-adapting to the human host (Vågene et al. 2022). However, these strains are not found in today's human populations anymore and they were probably replaced by *M. tuberculosis* L4 strains brought by the European colonists and subsequent waves of European migration to the Americas (Brynildsrud et al. 2018).

Additional aDNA analyses of 18^th^-century samples from Hungary placed the MRCA of L4 strains in the late Roman period, whereas the established mutation rate is in concordance with the above-mentioned estimation that MTBC emerged in the Neolithic (Kay et al. 2015). Analysis of well-preserved aDNA isolated from a calcified lung nodule of a 17^th^-century Swedish bishop is also consistent with Neolithic MTBC emergence (Sabin et al. 2020). However, it is important to emphasize that neither we can be certain of the constancy of the substitution rate during long periods of time and across lineages, nor we can fully understand the effects of latency on the evolution of MTBC (Gagneux 2018). Thus, although there is currently no consensus about the date of emergence of the MRCA of MTBC, it is probable that the evolution of the tubercle bacillus is much more complex and that the MTBC that we know today might be the result of one of several bottlenecks and selective sweeps (Smith et al. 2009). Therefore, more data coming from aDNA spanning different epochs will certainly help to get further insights into the detailed evolution of the MTBC.

### Mechanisms driving the mycobacterial evolution

Evolution of *M. tuberculosis* and mycobacteria in general, may involve different mechanisms of genetic transfer, which will be described and discussed in the two final sections of this review. Whereas it is thought that the recent evolution of the MTBC has likely been driven by clonal expansion from an *M. canetii*-like progenitor, mainly involving vertical gene transfer, mutation and gene loss events, the more distant evolution of the tubercle bacilli seems to have been strongly shaped by additional HGT episodes. Indeed, one hallmark of evolution lies in the capability of organisms to undergo HGT, which results in the exchange of genetic information that occurs independently of the inheritance from parent to offspring. HGT in bacteria takes mainly place via transformation, transduction, and conjugation, but may also include additional transfer routes such as extracellular vesicles, nanotubes, and gene transfer agents (Arnold et al. 2022). Whereas transformation and transduction do not require direct contact between donor and recipient bacteria, conjugation involves physical cell-to-cell interaction.

In regards to mycobacteria, the fast-growing *Mycobacterium smegmatis* has been a prominent study model to decipher the mechanisms of HGT in the genus. Pioneering work starting in the 1970s provided the first evidence of HGT in *M. smegmatis*, thus describing a conjugative-like genetic transfer (Mizuguchi and Tokunaga 1971, Tokunaga et al. 1973, Mizuguchi et al. 1976). The demonstration that the HGT process in *M. smegmatis* corresponds to a plasmid-independent conjugal transfer was achieved two decades later via mating assays between several pairs of strains, each strain carrying a chromosomally encoded antibiotic resistance, generating double-resistant recombinants following extended overnight co-culture on solid media (Parsons et al. 1998). As this transfer could originate from multiple *cis*-acting initiation sites while requiring sufficient homologous sequence at both ends to be successful (Wang et al. 2003, Wang and Derbyshire 2004), and since it results in unidirectional transmission from a donor strain towards a recipient strain of multiple, unlinked, and randomly spread chromosomal DNA fragments, the term of DCT was finally introduced (Gray et al. 2013). Therefore, the conjugative ability of mycobacteria differs from the other types of bacterial conjugation by the genome-wide mosaicism that can be seen in the mating progeny (Derbyshire and Gray 2014, Gray and Derbyshire 2018). Although DCT has for a long time only been demonstrated experimentally in *M. smegmatis*, other NTM widely spread within the phylogenetic tree of the genus—e.g. *Mycobacterium abscessus* (Sapriel et al. 2016), *M. avium* (Yano et al. 2017, Bannantine et al. 2020), and *M. kansasii* (Tagini et al. 2021)—depict such mosaic genome structure and organization, thus suggesting that this type of genetic transfer contributed to shape mycobacterial evolution at a large scale.

Regarding the tubercle bacilli, their ability to undergo DCT was a matter of debate. Given the recombinogenic population structure that is found in the *M. canettii* clade, and which likely corresponds to the condition that existed prior to the evolutionary bottleneck undergone by the MTBC members during their clonal expansion, it was assumed that DCT occurred in the genus until *M. canettii* emerged, but then became disabled in the last common ancestor of the MTBC (Gutierrez et al. 2005, Supply et al. 2013, Mortimer and Pepperell 2014). A genetic marker highlighting the ability of *M. canettii* for HGT is represented by the CRISPR/Cas locus. *Mycobacterium canettii* strains A and D harbor a very similar type III-A CRISPR/Cas system to the one that is found in the MTBC, whereas other *M. canettii* strains are characterized by type I systems with variable subtypes (He et al. 2012, Supply et al. 2013, Singh et al. 2021, Brenner and Sreevatsan 2023). By contrast, evaluating genetic transfer within the MTBC is challenging due to the high similarity in their genome sequence (over 99.9% nucleotide identity). In some studies, it was suggested that this high sequence similarity might prevent to detect rare homologous recombination events (Namouchi et al. 2012, Patané et al. 2017, Reis and Cunha 2021). However, the results of these studies are in conflict with several other studies that (*i*) either could not identify relevant recombination rates within the MTBC or if so, at a too weak level to have a sufficient impact on genetic diversity (Chiner-Oms et al. 2019); (*ii*) find no evidence to associate drug resistance acquisition with gene transfer in *M. tuberculosis* (Xia 2023); or (*iii*) link such putative recombination signals to low-quality sequencing data and spurious read alignments or assemblies (Godfroid et al. 2018). In any case, the questions whether tubercle bacilli were still capable of undergoing DCT and whether such an ability was maintained up to the MTBC branching point, remained an open and debatable question until recently. In 2016, a proof-of-concept article based on mating assays between selected *M. canettii* strains described the first experimental evidence that DCT was still active in certain tubercle bacilli. DCT was identified (*i*) between the *M. canettii* strain A carrying an integrative plasmid with a hygromycin-resistance marker used as donor strain; and (*ii*) the *M. canettii* strain L carrying a non-mobilizable plasmid providing resistance to kanamycin, used as recipient strain (Boritsch et al. 2016b). Recombinants that resulted from the extended contact between both strains were resistant to both antibiotics and depicted a genome mosaicism structure typical of DCT seen in rapidly growing mycobacteria, where multiple genomic regions from the *M. canettii* A donor strain were detected at random locations along the recombinant *M. canettii* L genome backbone. These initial results were extended in a recent study reporting that the ability for chromosomal DNA transfer was not restricted to *M. canettii* strains, but rather a common feature of both *M. canettii* clade members and MTBC members (Madacki et al. 2021). Indeed, by using the *M. canettii* strain L as recipient and by improving certain mycobacterial culture conditions, a large variety of recombinants were obtained independently of using *M. canettii* or MTBC strains as donors (Table 1). By contrast, no recombinants were found when using *M. kansasii* or *M. lacus* as donor strains, which might be due to reduced efficiency for homologous recombination linked to the larger phylogenetic distance between NTM and tubercle bacilli. Since all aforementioned mating experiments were performed with *M. canettii* strain L as recipient, one could ask whether other tubercle bacilli also retained an ability to act as recipient and to integrate foreign chromosomal DNA into their genome via DCT. When this question was evaluated with a large panel of MTBC and *M. canettii* strains, experimental evidence of successful conjugative transfer could be only seen when *M. canettii* strains L, G, or I were used as recipient strains (Table 1) (Madacki et al. 2021). These findings thus suggest that the sharing of genetic information is globally maintained in tubercle bacilli but that the ability to receive this information is restricted to a few *M. canettii* strains. It is thus tempting to speculate that DCT played an important role in shaping mycobacterial evolution within ancestral populations of tubercle bacilli, including strains of the *M. canettii* clade, as originally suggested by its recombinogenic population structure, but that the ability to receive and recombine foreign DNA into the genome has been lost over the course of evolution in most tubercle bacilli, favoring the clonal emergence of the members of the MTBC, including *M. tuberculosis* as a key pathogen.

**Table 1:**
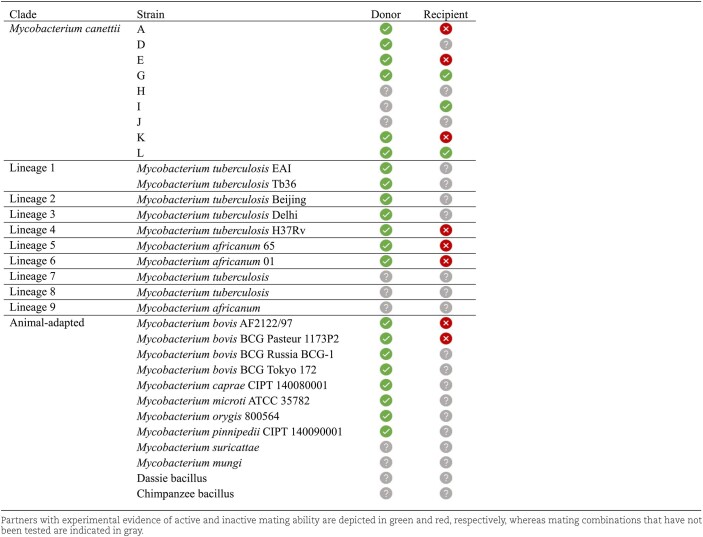
Donor and recipient mating identity among tubercle bacilli.

### ESX/type VII secretion systems as putative drivers of DCT

In mycobacteria, ESX/type VII secretion systems are encoded by up to five genomic loci (*esx1*-*5*) and correspond to molecular nanomachines located within the inner membrane of the complex mycobacterial cell envelope that serve for the transport of various ESX and ESX-associated substrates (Beckham et al. 2017, Famelis et al. 2019, Poweleit et al. 2019, Bunduc et al. 2021). While ESX secretion systems have often been linked to virulence-related tasks in pathogenic mycobacteria (Gröschel et al. 2016), we focus here on ESX-1 and ESX-4 systems as they have also been described to mediate conjugal DNA transfer in *M. smegmatis*. Indeed, ESX-1 was shown to negatively regulate transfer efficiency in the donor since it induced a hyper-conjugative phenotype upon disruption via transposon insertion in various *M. smegmatis* mutants used as donors (Flint et al. 2004). By contrast, deficiency of ESX-1 in the recipient strain appeared to prevent conjugal DNA transfer, indicating an essential role of the ESX-1 secretion system in the process of DNA acquisition and/or integration during DCT (Coros et al. 2008). It was thus hypothesized that proteins secreted by the ESX-1 machinery could either coat the donor cell surface to prevent physical contact with the recipient strain or act as signaling molecules to initiate or repress (depending on which partner is considered) mating (Derbyshire and Gray 2014). Given that the ESX-1 machinery is fundamentally identical between both donor and recipient strains, it is puzzling to interpret how it would function differently in each conjugal partner and, more generally, how it is defined which partner acted as donor and which one acted as recipient during DCT. By opposition to *oriT*-mediated Hfr conjugal transfer in which transconjugants can still receive but not transmit DNA anymore, it was shown in *M. smegmatis* that a subset of recombinants resulting from DCT maintained their ability to play the role of donor in a second mating assay, thus indicating that mating identity is genetically encoded (Wang et al. 2005). A genome-wide association study (GWAS) performed in *M. smegmatis* on F1 hybrid recombinants then suggested a link between the so-called *mid* locus and the donor mating identity (Gray et al. 2013). This locus, spanning from genes *MSMEG*_0069-*MSMEG_0071* and *MSMEG*_0076-*MSMEG_0078*, is encompassed within the *esx-1* locus of *M. smegmatis* and requires to be maintained to preserve the ability to function as a donor during DCT. However, a recent study redefined the role of the *mid* locus in conferring donor and recipient self-identity, as it rather appears that all strains with an intact or mutated *mid* locus can act as donor if the recipient possesses a compatible *mid* locus (Clark et al. 2022). By opposition, if both mating partners have an identical *mid* locus, they will be unable to undergo DCT. The *mid* locus, in particular the highly polymorphic gene *MSMEG_0070*, thus intervenes in kin recognition by establishing the donor–recipient pair compatibility and by extension whether mating can occur or not between two strains.

While the implication of the ESX-1 secretion system during DCT has been demonstrated for *M. smegmatis*, a study on HGT involving *M. canettii* and MTBC strains revealed that the ESX-1 system does not play an apparent role in DNA transfer in tubercle bacilli, neither in the donor nor in the recipient strain (Madacki et al. 2021). This conclusion was made based on a set of mutants used in the study. Indeed, modifying the donor and/or recipient strains either by mutating the *eccD1* gene, by deleting the ESX-1-encoding region, or by using natural ESX-1-deficient members of the MTBC (*M. microti* and *M. bovis* Bacille Calmette Guérin (BCG)) and their ESX-1-complemented counterparts, DCT could still occur normally and at a similar transfer efficiency as when using WT donor and/or recipient strains (Madacki et al. 2021). Those results thus contrast with the hyperconjugative phenotype and conjugative deficiency observed in *M. smegmatis* when disabling the ESX-1 machinery in the donor and the recipient, respectively, but are consistent with the weak conservation of the *mid* locus and the absence of *MSMEG_0070* ortholog among tubercle bacilli (Boritsch et al. 2016b). These observations and conclusions are also in line with the fact that several NTM species show evident recombinogenic population structures, but lack ESX-1 secretion systems, as for example the slow-growing species *M. avium* (Yano et al. 2017), or the fast-growing species *M. abscessus* (Sapriel et al. 2016) suggesting that ESX-1-independent DCT is widely spread among fast-growing and slow-growing mycobacterial species and hence might have played and/or still play a major role in shaping the evolution of many mycobacterial species, including key human pathogens such as the tubercle bacilli.

Apart from the ESX-1 machinery, the ESX-4 secretion system appears to play a critical role in conjugal DNA transfer in *M. smegmatis* as well. The *esx-4* locus is considered as the most ancestral and the progenitor of all other *esx* loci since it is the only one found thus far across the phyla of Actinomycetota (Actinobacteria) and Bacillota (Firmicutes), whereas the other ESX systems (ESX-1, 2, 3, and 5) are restricted to mycobacteria (Gey Van Pittius et al. 2001, Gey van Pittius et al. 2006, Dumas et al. 2016, Newton-Foot et al. 2016, Mortimer et al. 2017). However, the ESX-4 secretion system has been considered for a long time to be non-essential and non-functional in almost all mycobacteria, notably because it lacks EccE4, which is one of the core internal components of the ESX nanomachine, thought to be necessary for its stability (Beckham et al. 2017, Famelis et al. 2019, Poweleit et al. 2019, Bunduc et al. 2021). *Mycobacterium abscessus* together with the closely related *Mycobacterium chelonae* and *Mycobacterium immunogenum* species are the only mycobacterial species identified thus far that possess a full *esx-4* locus including the *eccE4* gene (Dumas et al. 2016, Newton-Foot et al. 2016, Laencina et al. 2018). This observation is also correlated with the secretion of the ESX-4 ESAT-6-like substrates EsxT and EsxU, which was detected in *M. abscessus* (Laencina et al. 2018, Lagune et al. 2022), but was absent in *M. marinum*, for example (Wang et al. 2022). Whereas the ESX-4 machinery appears to be involved in the virulence and intracellular survival of *M. abscessus* by regulating phagosomal membrane rupture and acidification (Laencina et al. 2018, Lagune et al. 2022, Bar-Oz et al. 2023), the function of the ESX-4 machinery in other mycobacterial species might be different and might not rely on the presence of EccE4 and secretion of EsxT/U. Several reports have recently associated the ESX-4 system with various biological processes in other mycobacterial species lacking EccE4 and secretion of EsxT/U. In *M. marinum*, deletion of the ESX-4 apparatus component *eccC4* resulted in an increased secretion of ESX-1 and ESX-5 substrates, suggesting that these three machineries are intimately linked (Wang et al. 2022). Indeed, the induction of phagocytosis and rearrangement of actin cytoskeleton observed in macrophages upon *eccC4* deletion appears to be a consequence of the enhanced secretion of the ESX-1 substrate EspJ. In addition, the localization and secretion of the exotoxin CpnT, which contains the TB necrotizing toxin (TNT) as C-terminal domain (Danilchanka et al. 2014), was shown to depend on the ESX-4 machinery in conjunction with other ESX systems in *M. marinum* (Izquierdo Lafuente et al. 2021) and *M. tuberculosis* (Pajuelo et al. 2021). In *M. smegmatis*, the ESX-4 secretion apparatus is essential for conjugation. While it had no apparent role in the donor strain, disruption of *eccC4* or *eccD4* in the *esx-4* locus by transposon insertion or gene deletion in the recipient strain abolished DCT (Gray et al. 2016). Consistent with this phenotype, physical contact during mating between donor and recipient partners highly induced the expression of *esxU/T* transcripts specifically in the recipient strain. This transcriptional activation of the *esx-4* locus, which appeared to be mediated by the alternative σ factor SigM (Clark et al. 2018), required a functional ESX-1 machinery in the recipient, but not in the donor strain, to occur. Given that disruption of either ESX-1 or ESX-4 in the recipient strain abrogates transfer in *M. smegmatis*, it suggests that both ESX-1 and ESX-4 secretion systems act in concert and are required during DCT and that the ESX-1 machinery intervenes upstream of the ESX-4 machinery in this fast-growing mycobacterial species. Since the ESX-1 apparatus was already demonstrated to have no role during DCT in tubercle bacilli (Madacki et al. 2021), it remains to be tested whether in these slow-growing and pathogenic mycobacterial species the ESX-4 machinery is involved or not in the process of HGT. On one hand, the ESX-4 system appears to be functional in *M. tuberculosis* as it was suggested to be involved in phagosomal membrane rupture (Pajuelo et al. 2021) and heme acquisition (Sankey et al. 2023). On the other hand, SigM also regulates positively the expression of *esxT* and *esxU* upon overexpression in *M. tuberculosis* (Raman et al. 2006, Agarwal et al. 2007, Rustad et al. 2014). Therefore, investigating whether the ESX-4 apparatus and SigM are implicated during DCT in tubercle bacilli is certainly worth of further investigation as it will shed light on this mechanism which might be important for driving mycobacterial evolution also in species other than *M. smegmatis*. Like other ESX systems, the ESX-4 system might represent a highly versatile macromolecular translocator that has adapted to fulfill different functions in various mycobacterial species, which may range from protein transport in *M. abscessus* to yet unknown functions required during HGT in other mycobacterial species.

## Conclusions

In conclusion, the recent availability of thousands of mycobacterial genome sequences and their analysis has confirmed and further enriched our understanding of the evolution of the tubercle bacilli. The recently added information on the species of the *M. tuberculosis*-associated phylotype together with new data on *M. canettii* HGT and experimental evolution as well as the discovery of novel MTBC lineages and aDNA samples provide exciting new insights into the molecular evolution of the tubercle bacilli and the various lineages of the MTBC and *M. tuberculosis sensu stricto* strains. Together with findings from recent TB susceptibility studies of the human host, these novel insights will also offer a better perception of the global TB epidemiology and emergence of new strain types.
